# A place-based analysis of COVID-19 risk factors in Bangladesh urban slums: a secondary analysis of World Bank microdata

**DOI:** 10.1186/s12889-021-10230-z

**Published:** 2021-03-15

**Authors:** Shaikh Mehdi Hasan, Susmita Das, Syed Manzoor Ahmed Hanifi, Sohana Shafique, Sabrina Rasheed, Daniel D. Reidpath

**Affiliations:** International Centre for Diarrhoeal Disease Research, Bangladesh, Health System and Population Studies Division, Mohakhali, Dhaka, 1212 Bangladesh

**Keywords:** COVID-19, Urban slums, WASH, Crowding

## Abstract

**Background:**

There is a lack of research investigating the confluence of risk factors in urban slums that may make them accelerators for respiratory, droplet infections like COVID-19. Our working hypothesis was that, even within slums, an inverse relationship existed between living density and access to shared or private WASH facilities.

**Methods:**

In an exploratory, secondary analysis of World Bank, cross-sectional microdata from slums in Bangladesh we investigated the relationship between intra-household population density (crowding) and access to private or shared water sources and toilet facilities.

**Results:**

The analysis showed that most households were single-room dwellings (80.4%). Median crowding ranged from 0.55 m^2^ per person up to 67.7 m^2^ per person. The majority of the dwellings (83.3%), shared both toilet facilities and the source of water, and there was a significant positive relationship between crowding and the use of shared facilities.

**Conclusion:**

The findings highlight the practical constraints on implementing, in slums, the conventional COVID19 management approaches of social distancing, regular hand washing, and not sharing spaces. It has implications for the management of future respiratory epidemics.

## Background

In January 2020, the COVID-19 appeared in the city of Wuhan, China marking the beginning of a global pandemic [1]. The disease is caused by the severe acute respiratory syndrome coronavirus 2 (SARSCoV2) which has person-to-person transmission qualities common to many respiratory viral infections [2].

The virus is picked up by the hands from contact with people and fomites, and transferred from the hands to the eyes, nose or mouth [3]. In confined spaces, it may be carried in aerosolized, speech droplets [4]. The prevalent public health messages about the prevention of transmission are consistent with our understanding of droplet infections (e.g., [5]).

- Wash hands regularly with soap and water or an alcohol-based hand sanitizer;
- Avoid touching eyes, nose and mouth;
- Cover your mouth and nose with your bent elbow or tissue when you cough or sneeze and then dispose of the used tissue immediately and wash your hands;
- Maintain at least 1 m (in some advice 2 m [6]) distance between you and others;
- Avoid crowded places;

There is additional advice for those who develop symptoms of COVID-19 infection or have been in close contact with someone with COVID-19. The additional advice focuses on physical isolation [7, 8].

- Do not leave your home for any reason;
- Remove vulnerable people from the household, and if that is not possible “stay away from them as much as possible”;
- Do not share towels;
- clean objects and surfaces you touch often;
- clean a shared bathroom each time you use it.

The combined messaging can be reduced to the need for (i) space, distance and isolation, (ii) water, sanitation and hygiene (WASH), and (iii) behavioral cough/sneeze etiquette. The third element of the message is broadly about individual behavior whereas the first two elements (space, distance and isolation, and WASH) are structural in nature. Structural elements are classically associated with a sociological literature around “structure and agency”, and the social determinants of health literature [9, 10]. An individual may intend to distance himself or herself and engage in appropriate hygiene but if the structural support is unavailable, the intention alone is insufficient.

Urban slums have been identified as potential hotspots for the spread of COVID-19, [11] and have been identified as reservoirs for the spread of other viral respiratory infections [12]. There is an obviousness to that identification: slums are crowded and poor, with a lack of access to basic utilities [13]. Notwithstanding the “self-evident” nature of the risk factors, there has been no quantification of those risk factors. Furthermore, slums are not homogenous urban spaces and the risk factors are unlikely to be distributed uniformly across the population of residents [14]. One might imagine, for instance, that those households that are more crowded are also less likely to have easy access to water for bathing and general hygiene.

The lack of empirical work in this area is unsurprising given the speed with which the pandemic has evolved. We identified only one review article of COVID-19 and slums, [15] and there were a few papers on preprint servers. None of the papers, however, utilized individual or household level data to quantify the presence of risk factors, or their co-occurrence.

In this research we examine the relationship between crowding and access to WASH facilities, with a working hypothesis that there is an inverse relationship such that more crowded living conditions are also associated with poorer access to WASH.

## Methods

A secondary analysis was conducted of the publicly available microdata from the World Bank’s Bangladesh “Urban Informal Settlements Survey 2016” [16, 17]. The cross-sectional, household survey was based on a sample of 600 households (588 responding households) in 63 urban slums. A single informant provided information for each household. The survey used a mixed sampling design ensuring the capture of data from small, medium and large slums. Unfortunately, the published microdata does not include sampling weights. An unweighted, model based analysis of the data is presented here.

In keeping with the strategies listed in the introduction for limiting the spread of the coronavirus, the analysis focuses on the number of people living in each dwelling, the degree of crowding, and whether toilet facilities and/or the source of water “for bathing, cooking and other purposes” (i.e., WASH) were shared amongst dwellings.

A simple, WASH score was developed according to the availability of shared or sole-use toilet facilities and shared or sole-use of water facilities, including water for bathing and general hygiene. The best WASH availability (i.e., the lowest score) was a dwelling with sole use toilet facilities and sole use of water for bathing. A moderate WASH score was associated with the shared use of either toilet or water sources, but not both. The highest WASH score was associated with shared toilets and bathing water sources. Crowding was calculated as the number of people living in a dwelling divided by the area (m^2^) of the dwelling.

The relationship between crowding and the WASH score was formally tested using a non-parametric, Kruskal-Wallis test and complemented with pairwise analyses using Wilcoxon’s test with a correction for the false discovery rate associated with multiple comparisons [18]. The median and 95% confidence intervals were reported where confidence intervals were estimated using a bias corrected and accelerated (*BCa)* bootstrap with 4000 resamples [19]. It is worth noting that a BCa confidence interval around a median will be asymmetrical if the distribution (as anticipated) is skewed. All analyses were conducted in the R statistical environment [20].

## Results

A total of 2516 people lived in the 588 surveyed dwellings. The median and modal number of people per dwelling was 4 and the mean was 4.3. Around 10% of residents were under 5 years of age (*n* = 261), and 5% were over 60 (*n* = 126) – a high risk category for COVID-19 fatality.

Most dwellings comprised a single room (*n* = 473, 80.4%), 15.1% (*n* = 89) were two-room dwellings, and 4.4% (*n* = 26) were dwellings of three or more rooms. The minimum dwelling size was 1.9 m^2^ and the maximum was (a seemingly implausible) 135.5 m^2^. The median dwelling size was 11.7 m^2^and the mean was 14.9 m^2^. Crowding within dwellings ranged from 0.55 m^2^ per person up to 67.7 m^2^ per person. The median density was 2.9 m^2^ per person and the mean 3.8 m^2^ per person. The US Army Public Health Command recommends a minimum of 6.7 m^2^/person in sleeping accommodation alone (i.e., barracks) [21]. Using this benchmark, 91.7% of the slum dwellings were all over-crowded, and more than 50% of the dwellings had less than half the recommended area per person.

**Fig. 1:**
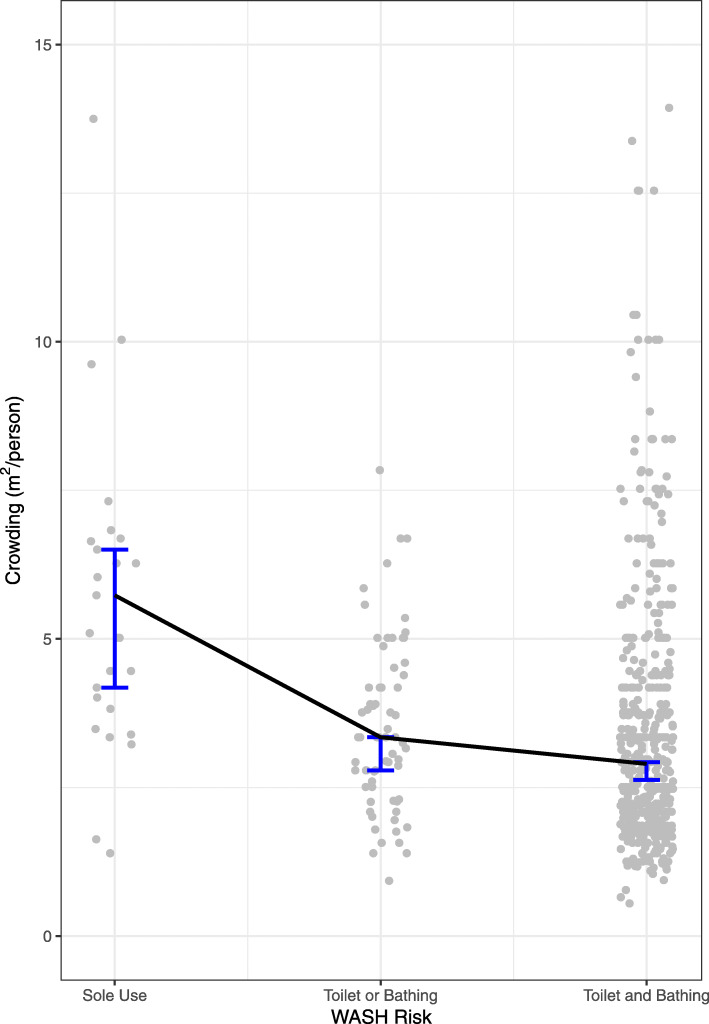
a plot of the level of crowding in dwellings according to the WASH score

Figure 1 shows a plot of the level of crowding in dwellings according to the WASH score. The median crowding and the bootstrap 95% confidence intervals are also shown. Six dwellings with, extreme values for crowding are excluded from the figure (though not the calculations of the medians or 95% confidence intervals).

There are a few observations to be made about the figure. First, the great majority of dwellings (*n* = 490, 83.3%) share both toilet facilities and the source of water for bathing, cooking and other purposes. Far fewer dwellings share either toilet or bathing facilities (but not both) (*n* = 69, 11.7%). Just under 5% of dwellings have sole use toilet and bathing facilities (*n* = 29, 4.9%). There, visually, appears to be a dose-response relationship between the WASH score and crowding. The median level of crowding is greatest in dwellings that use both shared toilet and bathing facilities (median = 2.9 m^2^/person, 95%CI: 2.63–2.93) followed by dwellings that share only toilet or bathing facilities (median = 3.34 m^2^/person, 95%CI: 2.79–3.34) with the least crowded dwellings being those that have sole use toilet and bathing facilities (median = 5.73 m^2^/person, 95%CI: 4.18–6.5). Dwellings with the highest WASH scores reported sharing facilities with a mean of 15.8 other dwellings and 79.3 other people. Dwellings with moderate WASH scores reported sharing facilities with a mean of 13.1 other dwellings and 60.4 other people.

The difference in the distribution of crowding for each of the WASH scores was formally tested using a Kruskal-Wallis test (chi-squared = 33.9, df = 2, *p* < .0001). Wilcoxon’s test, pairwise comparisons were made between sole use and shared toilet and bathing facilities (*p* < .0001), sole use and shared toilet or bathing facilities (*p* < .0001), and shared toilet and bathing facilities, and shared toilet or bathing facilities (*p* = .022).

## Discussion

Slums are by their very nature informal spaces, lacking enforced building codes [22]. It is hardly surprising, therefore, to learn that Bangladesh slums are crowded spaces with poor washing and toileting facilities. It is important to note, however, that the risk factors co-varied. The more crowded the dwelling, the more likely that WASH was also shared; i.e., being at greater risk of infection from one factor is associated with being at greater risk from another factor.

There is no, single, public health recommendation about the maximum level of crowding that is acceptable to limit the spread of droplet infections. Space alone cannot be the determining factor. Ventilation will affect the degree to which aerosolized particles may be hazardous. Evidence suggest that ventilation affects the spread of diseases like measles, chicken pox, influenza, tuberculosis, small pox, SARS and SARS-CoV-2 [23]. For diseases like influenza, airborne transmission occurs in two ways. First, through relatively large respiratory fluid droplets (10^1^–10^2^ μm) that settles on surfaces due to gravitational pull and requires close proximity for transmission. Second, finer particles (≤10 ^1^ μm) remain aerosolized and can be transmitted further away from the immediate vicinity of the infected person. For the latter source of infection, adequate ventilation is an important strategy to limit outbreaks [24]. Although the importance of the relative contribution of different modalities of infection for COVID-19 remains to be fully identified, limiting airborne transmission through improvement of ventilation, appropriate building engineering are thought to be important, along with the maintenance of regular hand washing and avoidance of shared spaces/facilities [25].. Around 627 million people worldwide share sanitation facilities [26]. As contact transmission is one of the important ways for the spread of the COVID-19 virus, infected taps, doors, knobs, toilets and handwashing surfaces can all effectively propagate the spread of the pandemic [26]. Furthermore, if there are peak times of use, as people from different households mix, the potential for aerosol transmission increases.

Prior research from slums in Bangladesh identified that nearly 82% of the slum households are single room dwellings and 48% possess a dwelling area of less than 9.3 m^2^ [27]. The current study suggests that more than 90% of slum dwellings are overcrowded, even according to the minimalist view of non-crowding adopted here. Moreover, the majority of the households possess WASH risk that makes them almost defenseless against COVID-19 infection. Data from the slums in Dhaka reported that toilet sharing (90%) is a very common phenomenon in the slums. Nearly 32% of the slum households shared their latrines with 10 or more households, 32.4% shared their latrines with 5–9 households and 26.2% with 1–4 households [27].. This level of vulnerability was similarly identified in previous work on social distancing in Indian slums [28]..

South Asia is home to some of the biggest slums in the world, with a substantial portion of its population living in these squalid arrangements of crowded, unhygienic conditions. COVID- 19 has the potential to spread across the slums of Mumbai (Dharavi), Karachi, Dhaka (Korail), and engulf the megacities housing them and beyond [29]. The pandemic has exposed the health security risks of the urban slums and will perhaps allow us to rethink urban development. History will show that slums were or were not implicated in the COVID-19 pandemic. This analysis, however, highlights the likely risks of slums as incubators and accelerators of infectious respiratory disease. In a recent national COVID-19 seroprevalence study, conducted by the Institute of Epidemiology Disease Control and Research (IEDCR) in collaboration with icddr,b, the seroprevalence of COVID-19 IgG and/or IgM was found to be 45% in Dhaka city and 74% in the slums [30].. Armed with this knowledge, it is important to consider the slum realities and rethink contextualized and novel short-term and long-term pandemic management approaches that focus on the intersections of urban planning and health service delivery improvement [31]. The contextual realities of slum are a complex mixture of numerous social, cultural and political factors that influence attitude and practice, and ultimately affect the success of any WASH related interventions [32].. In Bangladesh, most of the slums are built on government land and rented out to the dwellers, who are usually rural-urban migrants. The informal nature of residency does not allow for public utility services like water and electricity.

Politics play an important role in these settlements both internally and externally. Politically well-connected people usually control the slums and the available services. Politics, poverty, marginalization and discrimination against poor neighborhoods also influences the maintenance or destruction of such informal settlements [32]. The ‘illegality’ and the ‘temporariness’ of the settlements and the associated insecurity shapes the relevant sanitation and hygiene practices of the slum dwellers [32]. The fact that Bangladeshis are ‘washers’ [use water for defecation and urination] also creates more pressure on the availability on the availability of these resources in the informal settlements [32].

In many low- and middle-income countries around the world this pandemic has provided an opportunity to rethink urban development with a renewed focus on infection control in slums. Examples include the provision of portable hand washing stations in Kenya and plans in Namibia to “decongest” informal settlements through to intensive screening and testing in Dharavi slums in Mumbai, [33]. Low cost, sustainable, effective interventions or strategies need to be devised in the light of the complex socio-spatial, community and governance dynamics of slums. For example, effectiveness of cheaper, acceptable local innovations like *soapy water* solution against COVID-19 should be evaluated [34].

The COVID-19 pandemic is not ending soon and it will not be the last infectious disease epidemic to sweep through a megacity. It would be wise for us to learn the painful lessons that it offers.

## Conclusion

Slums pose an intricately woven set of health impediments that make it challenging to mount an appropriate response to the COVID-19 pandemic. They represent a confluence of informality, overcrowding, and poor infrastructure all of which increase the risk of exposure to COVID-19 [35]. Slums are also a significant source of low cost labour for megacities and can, therefore, become accelerators and incubators of epidemics [35]. Conventional containment measures don’t work within slum settings increasing their potential effect [35]. COVID-19, nonetheless, has provided an enormous, positive opportunity to rethink solutions to the entangled, ‘wicked problem’ of urban slums in epidemic control [36].. It has also implications for the management of future respiratory epidemics.

## Data Availability

The data used for this secondary analysis are publicly available in the World Bank Micro data library. Link: https://microdata.worldbank.org/index.php/catalog/2864

## Abbreviations

icddr,b: International Centre for Diarrhoeal Disease Research
SARSCoV2: Severe acute respiratory syndrome coronavirus 2
SARS: Severe acute respiratory syndrome
SIDA: Swedish International Development Cooperation Agency
UK: United Kingdom
WASH: Water sanitation and hygiene
